# Physiologic Range of Myocardial Mechano-Energetic Efficiency among Healthy Subjects: Impact of Gender and Age

**DOI:** 10.3390/jpm12060996

**Published:** 2022-06-18

**Authors:** Francesco Ferrara, Valentina Capone, Filippo Cademartiri, Olga Vriz, Rosangela Cocchia, Brigida Ranieri, Monica Franzese, Rossana Castaldo, Antonello D’Andrea, Rodolfo Citro, Salvatore Chianese, Roberto Annunziata, Flavio Marullo, Mario Siniscalchi, Marianna Conte, Chiara Sepe, Renato Maramaldi, Salvatore Rega, Giuseppe Russo, Massimo Majolo, Eliana Raiola, Andrea Salzano, Ciro Mauro, Bruno Trimarco, Raffaele Izzo, Eduardo Bossone

**Affiliations:** 1 Heart Department, University Hospital of Salerno, 84131 Salerno, Italy; fferrara1975@gmail.com; 2 Division of Cardiology, A Cardarelli Hospital, 80131 Naples, Italy; caponevalentina92@libero.it (V.C.); rosangela.cocchia@aocardarelli.it (R.C.); sasichian@gmail.com (S.C.); annunziata.robert@gmail.com (R.A.); flavio.marullo@gmail.com (F.M.); mario.siniscalchi@aocardarelli.it (M.S.); marianconte@hotmail.it (M.C.); makida@alice.it (C.S.); renatomaramaldi@gmail.com (R.M.); ciro.mauro1957@gmail.com (C.M.); 3 Department of Advanced Biomedical Sciences, “Federico II” University of Naples, 80131 Naples, Italy; trimarco@unina.it (B.T.); rafizzo@unina.it (R.I.); 4 Department of Radiology, Fondazione G. Monasterio CNR-Regione Toscana, 56124 Pisa, Italy; filippocademartiri@gmail.com; 5 Echocardiography Department, Heart Centre, King Faisal Specialist Hospital & Research Centre, Riyadh 11211, Saudi Arabia; olgavriz@yahoo.com; 6 IRCCS SYNLAB SDN, Via Emanuele Gianturco, 113-80143 Naples, Italy; brigida.ranieri@synlab.it (B.R.); monica.franzese@synlab.it (M.F.); rossana.castaldo@synlab.it (R.C.); andre.salzano@gmail.com (A.S.); 7 Department of Cardiology and Intensive Coronary Unit, “Umberto I” Hospital, 84014 Nocera Inferiore, Italy; antonellodandrea@libero.it; 8 Heart Department, University Hospital “San Giovanni di Dio e Ruggi d’Aragona”, 84125 Salerno, Italy; rodolfocitro@gmail.com; 9 Department of Translational Medical Sciences, Federico II University, 80131 Naples, Italy; salreg25@gmail.com; 10 Health Management Office, Antonio Cardarelli Hospital, 80131 Naples, Italy; direzione.sanitaria@aocardarelli.it (G.R.); massimo.majolo@aocardarelli.it (M.M.); eliana.raiola@aocardarelli.it (E.R.)

**Keywords:** myocardial mechanical efficiency, indexed myocardial mechanical efficiency, echocardiography

## Abstract

Background: Myocardial mechano-energetic efficiency (MEE) is the capability of the left ventricle (LV) to convert the chemical energy obtained from the cardiac oxidative metabolism into mechanical work. The aim of present study was to establish normal non-invasive MEE and MEEi reference values. Methods: In total, 1168 healthy subjects underwent physical examinations, clinical assessment, and standardized transthoracic echocardiographic (TTE) examination. MEE was obtained by TTE as the ratio between stroke volume (SV) and heart rate (HR): MEE = SV/HR [HR expressed in seconds (HR/60)]. Because MEE is highly related to left ventricular mass (LVM), MEE was then divided by LVM with the purpose of obtaining an estimate of energetic expenditure per unit of myocardial mass (i.e., indexed MEE, MEEi, mL/s/g). Results: The mean values of MEE and MEEi in the overall population were 61.09 ± 18.19 mL/s; 0.45 ± 0.14, respectively. In a multivariable analysis, gender, body surface area (BSA), diastolic blood pressure, left atrial volume indexed to BSA, E/e’ and tricuspid annular plane systolic excursion (TAPSE) were the independent variables associated with MEE, while age, gender, BSA and TAPSE were the independent variables associated with MEEi. Conclusions: The knowledge of age- and gender-based MEE and MEEi normal values may improve the global assessment of LV cardiac mechanics and serve as a reference to identify phenotypes at high risk of cardiovascular events.

## 1. Introduction

Myocardial mechano-energetic efficiency (MEE) is defined as the capability of the left ventricle (LV) to convert the chemical energy obtained from the cardiac oxidative metabolism into mechanical work [1]. It has been hypothesized that the increased energy expenditure relative to work contributes to disease progression [1]. In fact, in the case of pathophysiological states, such as heart failure, MEE is reduced [1]. However, although MEE can be quantified by dual-sided heart catheterization and selective catheterization of the coronary sinus, its widespread clinical applications have been limited by the need of invasive measurements requiring complex calculations [1]. In this regard, the availability of a surrogate measure of MEE based on a non-invasive echocardiographic approach allows more extensive clinical applications [2,3,4,5,6,7,8]. Indeed, strong evidence exists supporting the role of non-invasively derived indexed MEE (MEEi) in predicting cardiovascular (CV) clinical outcomes, with altered values associated with CV risk factors/disease such as insulin resistance and diabetes, hypertension, obesity, heart failure with preserved ejection fraction (HFpEF) [2,3,4,5,6,7]. Therefore, the aim of the present study was to explore the full range of MEE and MEEi values (as calculated by standard transthoracic Doppler echocardiography (TTE)) in a large cohort of healthy subjects, evaluating clinical and echocardiographic correlates.

## 2. Materials and Methods

### 2.1. Study Population

The study population consisted of 1168 healthy subjects (volunteers or subjects undergoing work ability assessment (mean age 43.4 ± 14.0); 123 (45.7%) men) referred to the echocardiographic laboratories of the Cardiology Division, “Cava de’ Tirreni-Amalfi Coast”, Heart Department, University Hospital of Salerno, Italy, and the Department of Cardiology and Emergency Medicine of San Antonio Hospital, San Daniele del Friuli, Udine, Italy [9,10]. The participants underwent full screening for CV disease including a questionnaire on medical history, use of medications, CV risk factors and lifestyle habits (alcohol intake, smoking, physical activity). Physical examinations (height, weight, heart rate (HR) and blood pressure (BP)) and clinical assessments were conducted according to standardized protocols by trained and certified staff. Body surface area (BSA) was calculated according to the DuBois formula (0.20247 × height (m) 0.725 × weight (kg) 0.425) [9]. Three BP measurements were obtained from the right arm by a sphygmomanometer, and the results were averaged to determine systolic and diastolic BP. Pulse pressure (PP) was calculated as systolic BP (SBP)—diastolic BP (DBP). The study was approved by the institution’s ethics board, and informed consent was obtained from all participants [9,10].

### 2.2. Echocardiography

A TTE with continuous ECG recording was performed with commercially available equipment on all subjects (Aloka α10—Aloka, Tokyo, Japan; Vivid 7—GE Healthcare, Milwaukee, WI, USA), according to the American Society of Echocardiography/European Association of Cardiovascular Imaging Guidelines, as previously described [11,12,13]. All studies were reviewed and analyzed off-line by two certified independent cardiologists, expert in TTE (F.F. and O.V.). Specific average measurements were taken of the 5 cardiac cycles. The left ventricular mass (LVM) (LVM indexed to body surface area (LVMI)) was estimated by left ventricular internal diameter (LVID), interventricular septum (IVS) and inferolateral wall thickness (PWT) at end-diastole from the parasternal approach, carefully obtained perpendicular to the left ventricular (LV) long axis: LVM = 0.8 × 1.04 × [(IVS + LVID + PWT)^3^ − LVID^3^] + 0.6 g. The left ventricular outflow tract (LVOT) diameter was measured in the zoom mode from the parasternal long axis view using standard electronic calipers in mid-systole, between the hinge points of the aortic valve leaflets from inner edge to inner edge. The LV stroke volume (SV) was calculated as the product of LVOT area and LVOT velocity time integral (VTI), obtained by a pulsed wave Doppler: SV = π × (LVOT/2)^2^ × LVOT VTI [14].

### 2.3. Myocardial Mechano-Energetic Efficiency Measurements

MEE can be defined as the ratio between the external systolic work and the total amount of energy produced by cardiomyocytes, estimated by the rate pressure product, which is an indirect measure of MVO2 [2,3,4,5]. LV MEE was estimated as the ratio between SW and MVO2 [2,3,4,5], SW as the product SBP × SV (mmHg × mL), and MVO2 using the “double product” (DP) of SBP in mmHg × HR, as the time of cardiac cycle (CC) by the following formula: CC = HR/60 in seconds (HR/60). Thus, MEE (mL/s) was calculated as follow: SBP × SV/SBP × HR = SV/HR, where HR was expressed in seconds (HR/60) [15].

As MEE is highly related to LVM, MEE was divided by LVM to obtain an estimate of energetic expenditure per unit of myocardial mass (i.e., indexed MEE, MEEi, mL/s/g) [3].

### 2.4. Statistical Methods

Continuous variables are expressed as means and standard deviations (SD). The data were tested for normality through the Shapiro–Wilk test. The Wilcoxon rank-sum test or t-test was used, as required, for comparisons of continuous variables between groups. Categorical variables are expressed as percentages and were compared using the chi- square test or the Fisher’s exact test. To compare more than two groups, the Kruskal–Wallis test was used. A two-tailed *p*-value less than 0.05 was considered significant. Holm’s correction was used for multiple hypothesis correction, if necessary. Spearman’s rank correlation was carried out for continuous variables to assess univariate associations. The variables were selected according to their clinical relevance. Multivariable linear regression analysis, including all variables that showed a significant correlation from the univariate analysis, was constructed to assess the independent associations of these variables with MEE and MEEi. Inter-observer agreement was tested with two independent observers remeasuring echocardiographic parameters in 20 randomly selected cases. Intra-observer variability was considered in 20 randomly selected cases by repeating the measurements on 2 occasions. The inter- and intra-observer variability were examined using both paired t tests and intraclass correlation coefficients (ICCs). An ICC >0.9 indicated excellent agreement. A statistical analysis was performed using R software (version 3.6.1, Vienna, Austria) [16].

## 3. Results

The demographic data of the study population are reported in Table 1. Compared with men, women had a lower weight, lower BSA and body mass index (BMI), lower BP and higher HR. The main echocardiographic parameters are reported in Table 2 [9].

The mean values of MEE and MEEi in the overall population were 61.09 ± 18.19 mL/s and 0.45 ± 0.14, respectively. The upper and lower limits (CI 95%) of normal MEE and MEEi were 62.13–60.05 and 0.46–0.44, respectively (Table 3).

### 3.1. MEE and MEEi according to Gender

**Figure 1:**
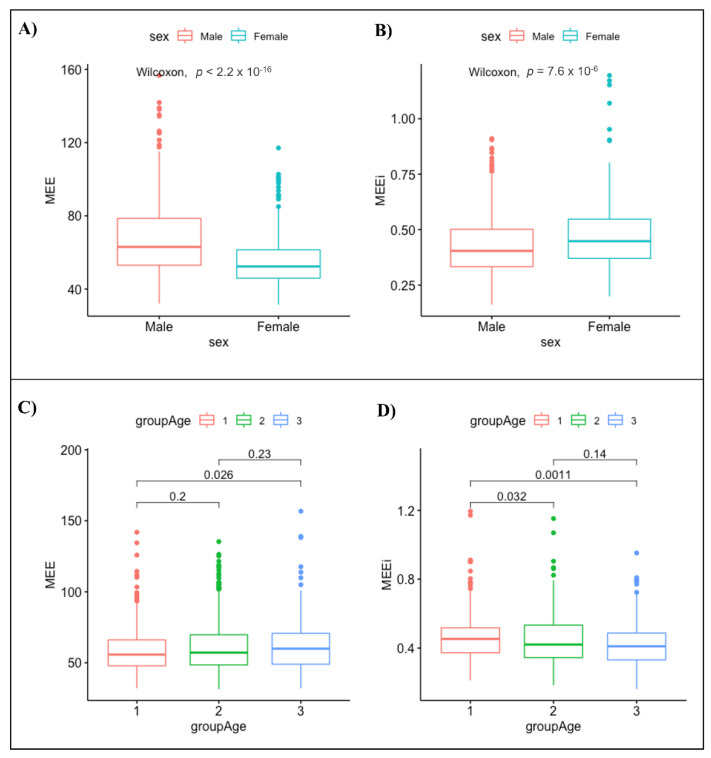
Normal ranges for MEE (**A**) and MEEi (**B**) by gender and normal ranges for MEE (**C**) and MEEi (**D**) by age.

The MEE values were higher in males than in females (67.27 ± 20.18 vs. 54.71 ± 13.13; *p*-value < 0.001) (Figure 1A). In contrast, MEEi was significantly higher in females than in males (0.47 ± 0.14 vs. 0.43 ± 0.13; *p*-value < 0.001) (Figure 1B). The upper limits and lower limits (CI 95%) of normal MEE and MEEi values for the male gender were 68.95–65.59 and 0.44–0.42, respectively. Conversely, the upper limits and the lower limits (CI 95%) of normal MEE and MEEi values for the female gender were 55.75–53.67 and 0.48–0.45, respectively (Table 3).

### 3.2. MEE and MEEi according to Age

**Figure 2:**
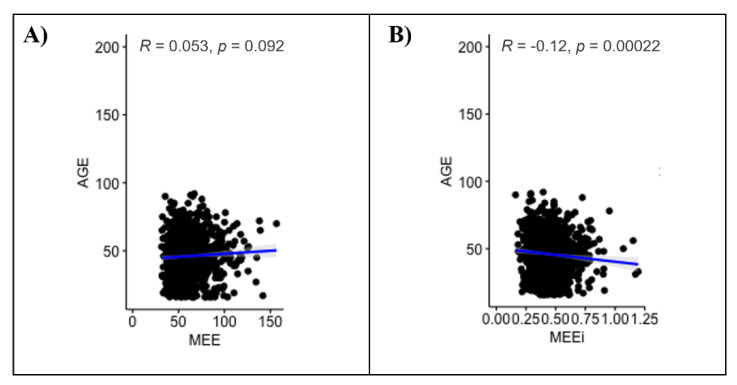
Univariate analysis of MEE (**A**) and MEEi (**B**) values by age.

No significant correlation between MEE and age (r = 0.053, *p* = 0.092) was found; conversely, a significant negative correlation between MEEi and age (r = −0.12, *p* value < 0.001) was found (Figure 2). The MEE values differed significantly only between the youngest (16–39 years, group 1) and the oldest age groups (>60 years, group 3) (*p* value = 0.026) (Figure 1C, Table 4). On the other hand, the MEEi values were significantly different between group 1 (16–39 years) and group 2 (40–59 years) (*p* value = 0.032) and between group 1 and group 3 (>60 years) (*p* value = 0.0011) (Figure 1D, Table 4).

### 3.3. Clinical and Echocardiographic Correlates of MEE and MEEi

**Figure 3:**
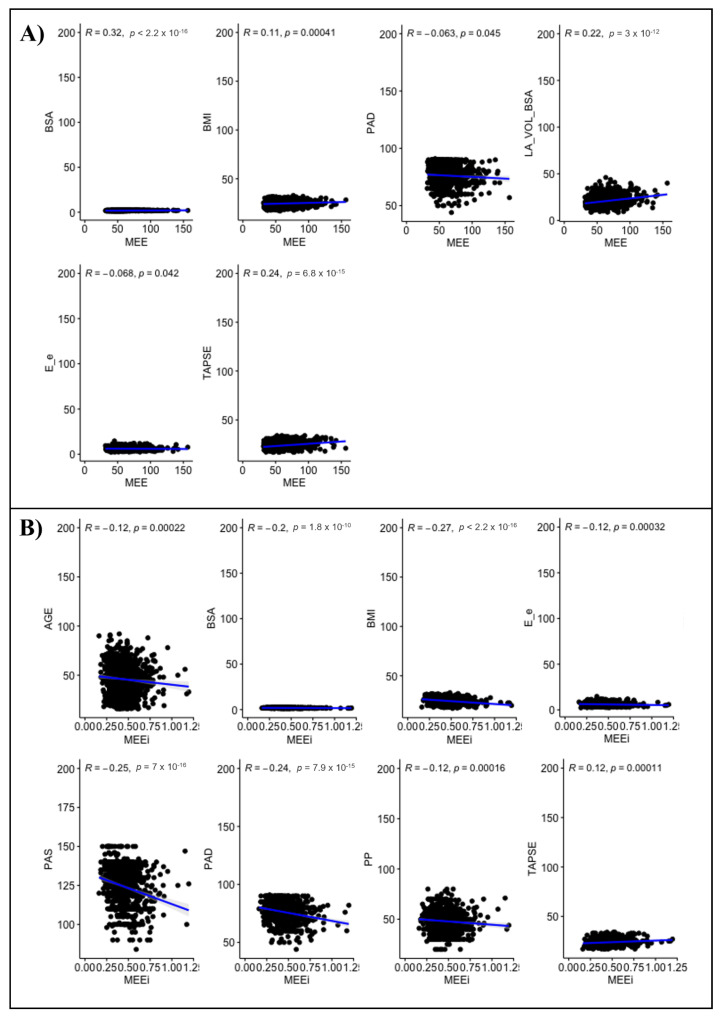
Univariate analysis of MEE (**A**) and MEEi (**B**) values.

In univariate analysis, MEE correlated negatively with gender (r= −0.345, *p*-value < 0.001) and positively with BSA (r = 0.32), BMI (r = 0.11), left atrial volume indexed to BSA (LAVI) (r = 0.22) and TAPSE (r = 0.24) (all *p*-values < 0.001). A Significant but weak and negative correlation between MEE and DBP (r = −0.063, *p* = 0.045), E/e’ (r = −0.068, *p* = 0.042) was found. No significant correlation between MEE and SBP, PP, mean blood pressure (MBP), left ventricular ejection fraction (LVEF), tricuspid regurgitation velocity (TRV) was seen in this sample of normal adults (all *p*-values > 0.05) (Figure 3A).

In a multivariable analysis, gender, BSA, DBP, LAVI, E/e’ and TAPSE were the independent variables associated with MEE (β coefficient −0.146, 0.219, −0.003, 0.009, 0.015 and 0.015 respectively; all *p*-values < 0.05) (Table 5).

In univariate analysis, MEEi correlated significantly and negatively with gender (r = −0.13) and with age (r = −0.12, *p* = < 0.001), BSA (r = −0.2), BMI (r = −0.27), SBP (r = −0.25), DBP (r = −0.24), PP (r = −0.12), MBP (r = −0.28), E/e’ (r = −0.12) and TAPSE (r = 0.12) (all *p*-values < 0.001). The MEEi values significantly correlated with gender (rho = −0.134, *p*-value < 0.001). No significant correlation between MEEi and LAVI, LVEF and TRV was found (all *p*-values > 0.05) (Figure 3B).

In a multivariable analysis age, gender, BSA and TAPSE were the independent variables associated with MEEi (β coefficient 0.002, −0.153, 0.205 and 0.019, respectively; all *p*-values < 0.05) (Table 6).

## 4. Inter- and Intraobserver Variability

The overall inter- and intra-observer agreement rates were similar, showing non-significant variability. The interobserver variability analysis revealed an ICCs of 0.96 (95% Confidence Interval: 0.94 to 0.98). The intraobserver agreement rates were 0.97 (95% Confidence Interval: 0.96 to 0.99).

## 5. Discussion

MEE provides useful information about LV cardiac mechanics, representing the capability of the LV to transform the chemical energy obtained from oxidative metabolism into mechanical work [1]. It appears an attractive simple tool that may improve CV risk stratification [17].

### 5.1. Previous Studies

Several investigators reported the MEE and MEEi values of subjects with CV risk factors, including arterial hypertension, obesity, diabetes, hyperlipemia and former or current smoking habits (Table 7).

De Simone et al. reported the MEE values of 255 subjects (F/M = 151/105; mean age = 35.3 ± 11.9 years) without CV risk factors, as a normotensive control group of 306 hypertensive patients (F/M = 129/177; mean age = 47.48 ± 10.45 years) free of CV disease [2]. The mean MEE values were not significantly different in normotensive controls vs. hypertensive patients (86.1 ± 25.7 vs. 85.4 ± 22.6 mL/s, *p* value ≤ 0.78). As a note, hypertensive patients with low LV mechanical efficiency (MEE values below the 90th percentile of the normal distribution) had much higher HR, systolic BPs, and pulse pressure than those exhibiting normal LV mechanical efficiency (all *p* < 0.001). Low MEE was also associated with inappropriately high LV mass (*p* < 0.0001).

Furthermore, among 12,353 hypertensive patients (F/M = 5429/7008; mean age = 52.4 ± 12.5 years), De Simone et al. showed that reduced MEE was associated with altered metabolic profile, LVH, and concentric LV geometry and independently predicted hard CV events, reducing the statistical impact of LVH [3].

The fat-associated CV dysfunction (FATCOR) study explored the association of MEEi with LV systolic circumferential and longitudinal myocardial function in 480 subjects with increased body mass index (BMI), without known CV disease (mean age 47 ± 9 years, 61% women, 63% obese, 74% with hypertension). Patients with lower MEEi values were more frequently men with obesity, hypertension, dyslipidemia, and a higher insulin resistance index (all *p* for trend < 0.05) [4]. The lower MEEi quartile (< 0.41 mL/s per g) was associated with lower circumferential and longitudinal LV myocardial function assessed by midwall fractional shortening (MFS) and global longitudinal strain (GLS), independent of cardiometabolic factors [4].

Interestingly, Losi et al., among 1912 unselected participants of a population-based cohort of American Indians with normal baseline EF, demonstrated that the lowest MEEi quartile (i.e., ≤0.34 mL × s^−1^ × g^−1^) predicted incident heart failure, after adjustment for LVH, prolonged relaxation and associated CV risk factors, including hypertension, obesity, diabetes, and smoking habits [5].

### 5.2. Uniqueness of the Present Study

To the best of our knowledge, this is the largest study that (a) comprehensively assessed the full range of MEE and MEEi values in a large cohort of 1168 healthy individuals stratified by age and gender; (b) demonstrated that the MEE values were higher in males than in females, whereas the MEEi values were significantly higher in females; (c) showed that the MEEi values were reduced in older age, while MEE was not significantly correlated with age; (d) revealed gender, BSA, DBP, LAVI, E/e’ and TAPSE as independent variables associated with MEE, and age, gender, BSA and TAPSE as independent variables associated with MEEi.

In the present study, the normal MEE values measured by the Doppler method were significantly lower than the values reported by De Simone et al. (61.0 ± 18.1 vs. 86.1± 25.7, respectively) in an older population (mean age = 45.4 ± 15.6 vs. 35.3 ± 11.9 years, respectively). In the latter study, SV was calculated as the difference between 2 D volumes (SV = LV end-diastolic volume—LV end-systolic volume), using the biplane method of disc summation (modified Simpson’s rule). This difference may also be consistent with the hypothesis that SV and CO are significantly lower if calculated by 2D rather than by Doppler and 3D methods (difference of 26 ± 0.4% of the measured 2D value) [19]. Thus, different methods (2D, Doppler and 3D) for calculating SV and thus the MEE and MEEi values should not be used interchangeably.

There are well-known gender differences in LV structure and function, including larger volumes, greater LVM, and higher CO and SV in men compared with women [9,19,20]. Similarly, the MEE values are significantly higher in males than in females. Thus, there is a need to adapt the reference value to the gender. In the present study, the lower limits of normal MEE and MEEi values were 65.5 mL/s and 0.41 mL/s/g in males and 53.6 mL/s and 0.45 mL/s/g in females (*p* value). These differences may be attributable to gender-related differences in biometrics characteristics. In this regard, a significant and independent association between the MEE and MEEi values and BSA was found.

Furthermore, our findings suggest a physiological impact of the aging process on MEE and MEEi (more evident), highlighting the need to adapt the normal reference values to age. This is consistent with previous results demonstrating that, in both genders, CO and SV tended to decrease with age [21]. Of note, the reduction in MEEi was related to an increase in LVM associated with aging. In contrast, as previously described, other LV function parameters such as LVEF and GLS in healthy subjects did not decrease significantly with older age, [9,22,23].

On the other hand, the significant and independent association of MEE and MEEi values with TAPSE may be indicative of the ventricular interdependence concept (the function of one ventricle is altered by changes in the filling of the other ventricle) [9].

## 6. Limitations of the Present Study

First, the study was limited to Caucasian healthy subjects. Thus, in the present study, the clinical relevance of MEE and MEEi for different races and pathologic states was not investigated. Secondly, additional echocardiographic techniques such as strain imaging and 3D echocardiography (3DE) were not performed.

## 7. Conclusions and Future Directions

We reported the physiologic ranges of MEE and MEEi measured by TTE in a large population of healthy subjects. MEE was significantly correlated with gender (higher values in males than in females) and BSA. Instead, MEEi was significantly decreased in older age and correlated significantly with gender (higher values in females than in males) and BSA. The knowledge of age-, BSA- and gender-based normal MEE and MEEi values may serve as reference to identify phenotypes at high risk of CV events.

## Figures and Tables

**Table 1:** Demographic and clinical characteristics of the study population.

Variable	Overall Population 1168 Patients (Mean ± SD) (Median)	Range	Women 613 (52.5%) (Mean ± SD)	Men 555 (47.5%) (Mean ± SD)	*p*-Value
Age (years)	45.4 ± 15.6 (46)	16–92	46.2 ± 15.4	44.6 ± 15.8	0.075
Height (cm)	168.6 ± 9.5 (168)	144–198	162.4 ± 6.7	175.5 ± 7.1	0.0001
Weight (Kg)	69.7 ± 12.0 (70)	41–113	62.7 ± 8.8	77.3 ± 10.4	0.0001
BMI (kg/m^2^)	24.4 ± 3.1 (24.2)	24.2–32.8	23.8 ± 3.24	25.1 ± 2.8	0.0001
BSA (m^2^)	1.79 ± 0.19 (1.78)	1.06–2.76	1.67 ± 0.14	1.92 ± 0.16	0.0001
SBP (mmHg)	123.9 ± 12.1 (125)	84–145	121.8 ± 12.7	126.1 ± 10.9	0.0001
DBP (mmHg)	76.1 ± 8.5 (78.5)	44–91	75.0 ± 8.4	77.2 ± 8.3	0.0001
MBP (mmHg)	92.0 ± 8.7 (93.3)	57.3–110.6	90.6 ± 8.9	93.5 ± 8.1	0.0001
PP (mmHg)	47.7 ± 9.7 (48)	20–80	46.8 ± 9.6	48.8 ± 9.7	0.001
HR (b/m)	71.1 ± 11.6 (70)	45–105	73.3 ± 10.8	68.7 ± 11.9	0.025

HR, heart rate; BMI, body mass index; BP, blood pressure; BSA, body surface area; DBP, diastolic blood pressure; MBP, mean blood pressure; PP, pulse pressure; SBP, systolic blood pressure; *p* values indicate sex-related differences.

**Table 2:** Echocardiographic parameters of the study population.

Parameters	Overall Population (Mean ± SD) (Range)	Women (Mean ± SD)	Men (Mean ± SD)	*p* Value
Septal wall thickness in diastole (mm)	8.6 ± 1.4 (6–11)	8.3 ± 1.3	9.0 ± 1.3	0.0001
Inferolateral wall thickness in diastole (mm)	8.6 ± 1.3 (6–11)	8.3 ± 1.2	9.0 ± 1.3	0.0001
LV end-diastolic diameter (mm)	47.3 ± 5.0 (36–58)	45.0 ± 4.2	49.8 ± 4.5	0.0001
LV EDV (mL)	80.5 ± 25.8 (41–158)	67.7 ± 18.0	94.6 ± 25.8	0.0001
LV ESV (mL)	29.4 ± 11.0 (11–72)	24.6 ± 7.9	34.7 ± 11.6	0.0001
LV EF (biplane) (%)	63.9 ± 5.6 (50–79)	64.1 ± 5.3	63.7 ± 6.0	0.226
LV mass index (g/m^2^)	77.0 ± 16.5 (38–145)	72.1 ± 14.9	82.7 ± 16.5	0.0001
LAVI (mL/m^2^)	20.4 ± 5.6 (8.5–46.0)	20.0 ± 5.2	20.8 ± 6.1	0.024
Mitral Peak E/e^’^ ratio	5.9 ± 1.7 (2.2–11.8)	6.1 ± 1.8	5.7 ± 1.7	0.0001
SVI (mL/m^2^)	38.7 ± 7.4 (22.0–73.1)	39.1 ± 6.7	38.3 ± 7.9	0.121
CI (L/min/m^2^)	2.7 ± 0.6 (1.73 −5.7)	2.8 ± 0.6	2.5 ± 0.6	0.0001
RV basal diameter (mm)	33.2 ± 3.6	32.0 ± 3.3	34.7 ± 3.3	0.0001
RV mid cavity diameter (mm)	25.8 ± 3.8	24.6 ± 3.2	27.3 ±3.9	0.0001
RV longitudinal diameter (mm)	63.8 ± 5.0	62.1 ± 4.1	65.4 ± 5.3	0.0001
RA major dimension (mm)	41.3 ± 4.6	39.9 ± 4.2	42.8 ± 4.6	0.0001
RA minor dimension (mm)	33.4 ± 4.4	31.9 ± 3.5	35.1 ± 4.6	0.0001
Tricuspid Peak E velocity (m/s)	0.51 ± 0.12	0.52 ± 0.11	0.50 ± 0.13	0.041
Tricuspid Peak A velocity (m/s)	0.37 ± 0.13	0.38 ± 0.13	0.36± 0.14	0.011
Tricuspid Peak E/A ratio	1.52 ± 0.61	1.501 ± 0.613	1.55 ± 0.607	0.196
TAPSE (mm)	23.6 ± 3.3	23.2 ± 3.0	24.1 ± 3.5	0.0001
SPAP (mmHg)	20.9 ± 5.9	21.2 ± 5.3	20.7 ± 6.5	0.143
TAPSE/SPAP	1.2 ± 0.4	1.1 ± 0.3	1.3 ± 0.5	0.0001
RVOT AT (m/s)	138.6 ± 17.5	139.6 ± 17.7	137.5 ± 17.1	0.062
PVR (WU)	1.3 ± 0.3	1.3 ± 0.3	1.3 ± 0.4	0.605

AT, acceleration time; BSA, body surface area; CI, cardiac index (CO/BSA); EDV, end-diastolic volume; EF, ejection fraction; ESV, end-systolic volume; LA, left atrium; LAVI, left atrial volume indexed to BSA; LV, left ventricular; PVR, pulmonary vascular resistance; RA, right atrial; RV, right ventricular; RVOT, right ventricular outflow tract; SD, standard deviation; SPAP, systolic pulmonary artery pressure; SVI, stroke volume indexed (SV/BSA); TAPSE, tricuspid annular plane systolic excursion; TDI, tissue Doppler imaging; TRV, tricuspid regurgitation velocity; TVI, time-velocity integral; *p* values indicate sex-related differences.

**Table 3:** MEE and MEEI values in the study population.

	(Mean ± SD)	Lower Limit	Upper Limit
MEE (mL/s)			
Overall	61.09 ± 18.19	60.05	62.13
Male	67.27 ± 20.18	65.59	68.95
Female	54.71 ± 13.13	53.67	55.75
MEEi (mL/s/g)			
Overall	0.45 ± 0.14	0.44	0.46
Male	0.43 ± 0.13	0.42	0.44
Female	0.47 ± 0.14	0.45	0.48

MEE, mechanical efficiency; MEEi, estimated energetic expenditure per unit of myocardial mass; SD, standard deviation.

**Table 4:** MEE and MEEI values stratified by age in the study population.

Variables	Age Subgroups (y)			*p*-Value	Intragroup *p*-Value
	16–39 years	40–59 years	>60 years		
	(Mean ± SD)	(Mean ± SD)	(Mean ± SD)		
MEE					
Overall	59.54 ± 17.44	61.41 ± 18.19	63.07 ± 19.33	0.086	C: 0.026;
Male	64.92 ± 18.74	68.32 ± 20.27	69.69 ± 22.56	0.15	
Female	52.59 ± 12.60	54.89 ± 13.01	57.35 ± 13.74	0.01	C: 0.0031;
MEEi					
Overall	0.46 ± 0.13	0.45 ± 0.14	0.43 ± 0.13	0.0045	A: 0.032; C: 0.001;
Male	0.44 ± 0.13	0.42 ± 0.14	0.42 ± 0.13	0.22	NS
Female	0.49 ± 0.14	0.47 ± 0.14	0.43 ± 0.14	<0.001	A: 0.037; B: 0.025; C: <0.001;

MEE, mechanical efficiency; MEEi, estimated energetic expenditure per unit of myocardial mass; NS, not significant; SD, standard deviation; y, yeas; intergroup A, age 16–39 vs. age 40–59; intergroup B, age 40–59 vs. age >60; intergroup C, age 16–39 1 vs. age >60.

**Table 5:** Multivariate analysis of THE MEE values.

	Estimate	Std. Error	*p*-Value
(Intercept)	0.417	0.158	0.008
Female	−0.146	0.024	<0.001
BSA	0.219	0.070	0.001
BMI	−0.003	0.003	0.331
DBP	−0.003	0.001	<0.001
LAVI	0.009	0.001	<0.001
E/e’	0.015	0.005	0.003
TAPSE	0.015	0.003	<0.001

BMI, body mass index; BSA, body surface area; DBP, Diastolic blood pressure; LAVI, left atrial volume indexed to BSA; TAPSE, tricuspid annular plane systolic excursion; *p* values indicate sex-related differences.

**Table 6:** Multivariate analysis of the MEEi values.

	Estimate	Std. Error	*p*-Value
(Intercept)	0.605	0.163	<0.001
AGE	0.002	0.000	0.002
Female	−0.153	0.024	<0.001
BSA	0.205	0.072	0.004
BMI	−0.004	0.003	0.243
SBP	−0.024	0.029	0.418
DBP	0.019	0.029	0.515
PP	0.023	0.029	0.426
E/e’	0.007	0.005	0.217
TAPSE	0.019	0.003	<0.001

BMI, body mass index; BSA, body surface area; DBP, diastolic blood pressure, PP, pulse pressure; SBP, systolic blood pressure; TAPSE, tricuspid annular plane systolic excursion; *p* values indicate sex-related differences.

**Table 7:** Previous studies providing MEE and MEEi values [2,3,4,5,6,7,8,18].

Study	N.	Gender (F/M)	BMI (kg/m^2^)	Age (y)	CVRF	EF (%)	MEE (mL/s)	MEEi (mL/s/g)	Remarks
De Simone G. et al. *Journal of Hypertension* 2009 [2]	255	151/105	27.1 ± 6.6	35.3 ± 11.9	Healthy subjects—no CVRF	64.6 ± 4.9	86.1 ± 25.7	-	Volunteers involved in a screening program of the department staff or subjects referred to the “Outpatient Nutrition Clinic”.
	56	26/29	27.9 ± 4.8	49.3 ± 9.5	Hypertension	63.1 ± 6.09	Low MEE	-	Subjects were divided in groups with normal and low myocardial mechanical efficiency (i.e., below the 90th percentile of the normal distribution; normal distribution: 85.4 ± 22.6) .After adjusting for age and sex, hypertensive patients with low MEE showed greater relative wall thickness and lower EF and midwall shortening than patients with normal MEE. Low MEE was also associated with inappropriately high LV mass.
	250	103/148	27.9 ± 4.3	47.1 ± 10.6	Hypertension	66.5 ± 5.4	Normal MEE	-	
De Simone G. et al. *Journal of Hypertension* 2016 [3]	12353	5429/7008	-	52.4 ± 12.5	Hypertension (100%), obesity (26%), diabetes (10%) *	66.3 ± 3.9	62.6 ± 14.4	F:0.35 ± 0.08 M:0.33 ± 0.07	Patients selected from the Campania Salute Network (CSN) Registry. Low MEE was associated with altered metabolic profile, LVH, concentric left ventricular geometry, and diastolic dysfunction and predicted CV end-points, independently of age, sex, LVH antihypertensive therapy, and CVRF.
Mancusi C. et al. *Journal of Clinical Medicine* 2021 [4]	111	F 42%	33 ± 5	48 ± 9	Hypertension (85%), obesity (75%), diabetes (10%) **	61 ± 6	-	≤0.41	Subjects participating in the fat-associated cardiovascular dysfunction (FATCOR) study. Reduced MEEi was associated with lower LV myocardial function both in the circumferential and in the longitudinal direction, independent of cardiometabolic factors.
	120	F 58%	32 ± 4	49 ± 9	Hypertension (76%), obesity (62%), diabetes (9%) **	62 ± 7	-	0.42–0.54	
	125	F 68%	32 ± 4	46 ± 9	Hypertension (71%), obesity (67%), diabetes (4%) **	64 ± 6	-	0.54–0.67	
	124	F 75%	31 ± 4	47 ± 9	Hypertension (65%), obesity (48%), diabetes (7.5%) **	63 ± 6	-	≥0.67	
Losi MA. et al. *Journal of Clinical Medicine* 2019 [5]	478	F 55%		60 ± 8	Hypertension (34%), obesity (58%), diabetes (57%), hyperlipemia (62%), former smoker (38%), current smoker (35%) *	-	-	≤0.34	Data from the “Strong Heart Study” (SHS), a population-based cohort with CVRF but free of CV disease. A low LV MEEi was a predictor of incident, non-AMI related HF in subjects with initially normal EF.
	479	F 65%		59 ± 8	Hypertension (29%), obesity (57%), diabetes (41%), hyperlipemia (59%), former smoker (36%), current smoker (34%) *	-	-	0.35–0.39	
	477	F 69%		60 ± 8	Hypertension (25%), obesity (51%), diabetes (37%), hyperlipemia (55%), former smoker (34%), current smoker (35%) *	-	-	0.40–0.44	
	478	F 68%		59 ± 8	Hypertension (22%), obesity (40%), diabetes (25%), hyperlipemia (57%), former smoker (33%), current smoker (39%) *	-	-	≥0.45	
Manzi MV. et al. *ESC Heart Fail.* 2022 [6]	5536	F 42.1%		53.40 ± 11.41	Hypertension (100%), obesity (24.3%), diabetes (9.8%), smoker (19.1%) *	65.8 ± 3.86	-	0.34 ± 0.07	Patients selected from the Campania Salute Network (CSN) Registry. Lower values of MEEi at baseline significantly contributed to identify patients more prone to develop LV systolic dysfunction.
	137	F 38%		59.46 ± 11.58	Hypertension (100%), obesity (27%), diabetes (18.2%), smoker (19%) *	65.2 ± 11.5	-	0.32 ± 0.08	
Bahlmann E. et al. *Open Heart* 2021 [18]	569	F 35%	27.9 ± 4.7	68 ± 10	Hypertension (88%), obesity (28%) ***	65 ± 7	-	<0.34–0.26 ± 0.06	Post hoc analysis performed within the prospective Simvastatin and Ezetimibe in Aortic Stenosis (SEAS) study. In patients with initially asymptomatic aortic stenosis, a low MEEi was associated with clustering of cardiometabolic risk factors, lower LV myocardial function and subsequent increased mortality during a 4.3 year follow-up, independent of known prognosticators.
	1134	F 41%	26.3 ± 4.1	67 ± 10	Hypertension (81%), obesity (16%) ***	67 ± 6	-	≥0.34–0.54 ± 0.16	
Fiorentino TV et al. *Diabetes Research* *and Clinical Practice* 2021 [7]	617 NGT 1 h-low (1)	389/228	29.4 ± 6.6	44 ± 13	≥1 cardio-metabolic risk factors ****	-	-	0.41 ± 0.11	The study cohort consisted of 1467 non-diabetic adult subjects participating in the CATAMERI study. Subjects with NGT 1 h-high, isolated IFG, and IGT had a raised myocardial oxygen consumption and a reduced MEE.
	210 NGT 1 h-high (2)	100/110	30.1 ± 5.9	49 ± 12	≥1 cardio-metabolic risk factors ****	-	-	0.38 ± 0.11	
	237 Isolated IFG (3)	94/143	30.3 ± 5.3	54 ± 11	≥1 cardio-metabolic risk factors ****	-	-	0.37 ± 0.10	
	403 IGT (4)	217/186	31.4 ± 5.9	54 ± 12	≥1 cardio-metabolic risk factors ****	-	-	0.35 ± 0.09	
Cioffi G. et al. *Journal of Hypertension* 2021 [8]	432	F 64%	26.0 ± 4.5	57 ± 12	Hypertension (46%), obesity (16%), diabetes (9%), hyperlipemia (56%), active smoker (34%)	66 ±7	-	0.35 ± 0.11	The study population consisted of 432 outpatients with established chronic inflammatory arthritis without overt cardiac disease, compared to 216 patients without chronic inflammatory arthritis. In patients with chronic inflammatory arthritis, a low-MEE was a powerful prognosticator of adverse CV events.
	216	F 58%	25.4 ± 4.3	59 ± 14	Hypertension (46%), obesity (16%), diabetes (9%), hyperlipemia (56%), active smoker (34%)	64 ± 9	-	0.45 ± 0.10	

The table collects the values of MEE and MEEi provided by previous studies. Most studies included subjects with different cardio-metabolic risk factors, except for one study of 255 healthy subjects [3]. BMI, body mass index; CV, cardiovascular; CVRF, cardiovascular risk factors; EF, ejection fraction; IFG, impaired glucose tolerance; MEE, myocardial mechanical efficiency; MEEi, indexed myocardial mechanical efficiency; NGT, normal glucose tolerance. _*_ Arterial hypertension was defined as office BP values at least 140 (SBP) and/or at least 90 mmHg (DBP) or when participants were taking antihypertensive medications. Obesity was defined as a BMI of at least 30 kg/m^2^. Diabetes was defined as fasting plasma glucose >125 mg/dL or current antidiabetic treatment. ** Hypertension was considered present if the 24 h ambulatory BP was elevated or if the participants reported the use of antihypertensive medications. Obesity was defined as BMI ≥30.0 kg/m2. Diabetes mellitus was considered present if fasting blood glucose ≥7 mmol/L, 2 h blood glucose ≥11.1 mmol/L after a 75 g oral glucose test, or glycated hemoglobin A1c ≥6.5%. *** Obesity was defined as body mass index ≥30 kg/m^2^. Hypertension was defined as history of hypertension or current antihypertensive treatment or elevated blood pressure at the baseline clinical visit. **** Cardio-metabolic risk factors included family history of diabetes, dysglycemia, hypertension, dyslipidemia, and overweight/obesity. Individuals were classified as having normal glucose tolerance (NGT) when fasting plasma glucose was < 100 mg/dL and 2 h post-load glucose was <140 mg/dL; isolated impaired fasting glucose (IFG) when fasting plasma glucose was 100–125 mg/dL and 2 h post-load glucose was <140; impaired glucose tolerance (IGT) when 2 h post-load glucose was 140–199 mg/dL in accordance with the ADA criteria. Individuals with NGT were further subdivided into two groups (NGT 1 h-low and NGT 1 h-high) using the 1 h plasma glucose cut-off of 155 mg/dL.

## Data Availability

The data that support the findings of this review are openly available in the References section.
